# Toward Efficient Health Data Identification and Classification in IoMT-Based Systems

**DOI:** 10.3390/s25195966

**Published:** 2025-09-25

**Authors:** Afnan Alsadhan, Areej Alhogail, Hessah A. Alsalamah

**Affiliations:** Information Systems Department, College of Computer and Information Science, King Saud University, Riyadh P.O. Box 145111, Saudi Arabia; aalsadhan@ksu.edu.sa (A.A.); halsalamah@ksu.edu.sa (H.A.A.)

**Keywords:** data governance, data identification and classification (DIC), HIPAA compliance, health data privacy, internet of medical things (IoMT), medical data security

## Abstract

The Internet of Medical Things (IoMT) is a rapidly expanding network of medical devices, sensors, and software that exchange patient health data. While IoMT supports personalized care and operational efficiency, it also introduces significant privacy risks, especially when handling sensitive health information. Data Identification and Classification (DIC) are therefore critical for distinguishing which data attributes require stronger safeguards. Effective DIC contributes to privacy preservation, regulatory compliance, and more efficient data management. This study introduces SDAIPA (SDAIA-HIPAA), a standardized hybrid IoMT data classification framework that integrates principles from HIPAA and SDAIA with a dual risk perspective—uniqueness and harm potential—to systematically classify IoMT health data. The framework’s contribution lies in aligning regulatory guidance with a structured classification process, validated by domain experts, to provide a practical reference for sensitivity-aware IoMT data management. In practice, SDAIPA can assist healthcare providers in allocating encryption resources more effectively, ensuring stronger protection for high-risk attributes such as genomic or location data while minimizing overhead for lower-risk information. Policymakers may use the standardized IoMT data list as a reference point for refining privacy regulations and compliance requirements. Likewise, AI developers can leverage the framework to guide privacy-preserving training, selecting encryption parameters that balance security with performance. Collectively, these applications demonstrate how SDAIPA can support proportionate and regulation-aligned protection of health data in smart healthcare systems.

## 1. Introduction

Over the last hundred years, the healthcare industry has undergone a profound shift, moving from a hospital-focused model to one centered around patients. This evolution has given rise to smart healthcare systems (SHSs), which leverage cutting-edge technologies like the Internet of Medical Things (IoMT), cloud computing, and artificial intelligence to make medical care more efficient and intelligent. The COVID-19 pandemic further accelerated the adoption of these solutions, as the need for remote patient-doctor interactions surged. For instance, the global SHS market, valued at USD 153.6 billion in 2021, is projected to reach USD 461.76 billion by 2029 [1].

Today, the IoMT (or Internet of Health Things, IoHT) stands as a cornerstone of SHSs. By integrating wearable sensors, mobile devices, and cloud-based analytics, it enables real-time patient monitoring, improving care accuracy while reducing costs. Experts predict that by 2026, there will be over seven million IoMT-connected devices worldwide [2], highlighting their critical role in remote patient management, emergency response, and chronic disease control.

Despite its advantages, the IoMT faces serious challenges, particularly concerning patient data security. The FDA revealed that 82% of healthcare institutions experienced cyber threats in 2020–2021, with 34% involving ransomware attacks [3]. Given the high sensitivity of medical data, breaches can erode patient trust and lead to severe consequences. As a result, the healthcare cybersecurity market is expected to grow to USD 32.9 billion by 2028 [4], underscoring the urgent need for robust data protection measures. In addition, the distribution of patient information over different systems causes many security and privacy issues. For medical devices that are intended to be used for remote communications for healthcare procedures, patients’ privacy is of the utmost importance, but the majority of IoMT devices are unable to adequately protect sensitive data privacy on their own due to their limited resources.

The sensitivity of health data and the need for privacy attracted IoMT researchers during and after COVID-19 for two reasons. First, IoMT users are vulnerable to privacy threats because of the distributed structure of IoMT systems and the weakness of the access and modification permissions of stored electronic health record (EHR) data in the shared central cloud server by unauthorized users. Second, data analysis on the cloud involves computing on servers owned by third parties who may sell the data to suspicious entities for several purposes like marketing and advertising.

According to the World Health Organization (WHO) [5], health data privacy refers to the right to control personal health information and make informed decisions about its use. Based on this general definition, we can define patient data privacy in IoMT systems as the patients’ right to control their personal and medical data that is collected, transmitted, processed, stored, or shared by IoMT healthcare systems. Many nations currently have laws in place controlling the gathering and storage of sensitive patient health data to ensure the data privacy, such as the US’s Health Insurance Portability and Accountability Act (HIPAA) and the EU’s General Data Protection Regulation (GDPR). In Saudi Arabia, the Saudi for Data and Artificial Intelligence Authority (SDAIA) has established regulations for data privacy protection [6]. The importance of IoMT privacy preservation lies in protecting sensitive patient data and maintaining trust within the healthcare ecosystem. It ensures that individuals have control over their personal health data and can decide who has access to them. This trust is essential for encouraging the adoption of innovative healthcare technologies and ensuring that patients are willing to share their personal health information.

In this paper, we address the privacy preserving challenge by proposing the SDAIPA (SDAIA-HIPAA) model, a hybrid classification standard designed to enhance IoMT security through systematic data identification and classification. Our approach aims to safeguard sensitive health information while ensuring compliance with regulations like HIPAA and GDPR, thereby strengthening the foundation of future e-healthcare systems. The contributions of this paper are as follows:

1. Conducting a review of existing methods in IoMT data identification and classification highlighting critical gaps in data privacy efficiency that motivate our approach.
2. Proposing SDAIPA (SDAIA-HIPAA) framework with two core components:
  - Standard Data Identification—Our model introduces a single and comprehensive data identification list to eliminates inconsistencies in detecting regulated data, ensuring compliance efficiency and interoperability across heterogeneous systems.
  - Robust Data Classification—We design a hybrid sensitivity classification model that integrates quantitative privacy risk assessment in align with HIPPA and SDAIA regulations to granular IoMT health data protection.

Validating the model’s effectiveness and accuracy through Delphi and expert elicitation methods and discussing its implications for the healthcare industry. Collectively, these contributions offer a structured foundation to support risk-aware IoMT data protection and guide encryption optimization.

A wide range of prior work has investigated IoMT data classification using different perspectives, including clustering, performance optimization, and detection approaches. Clustering-based methods have been used to group and analyze health-related data attributes, whereas performance-oriented research has aimed at improving system efficiency, scalability, and resource allocation in IoMT environments. In parallel, detection-oriented studies have concentrated on identifying anomalies, intrusions, or irregular patterns to strengthen IoMT security. Although these works highlight important directions, they do not directly address the challenge of sensitivity-aware classification, which is central to safeguarding patient privacy and ensuring compliance with evolving regulations. In contrast, the SDAIPA framework introduced in this study explicitly builds on this perspective by integrating regulatory principles with structured sensitivity scoring to provide a more privacy-focused foundation for IoMT data management.

The organization of this paper is as follows. Section 2 reviews related work, providing context for our research. Section 3 presents the SDAIPA (SDAIA-HIPAA) Identification and Classification Model, including its methodology, implementation and validation. Section 4 highlights practical considerations for implementing the SDAIPA framework in real-world Smart Healthcare Systems (SHS). Finally, Section 5 concludes the paper with a discussion of key findings and potential future directions.

## 2. Related Work

Data identification and classification process are the cornerstone of effective data management. By categorizing data based on its sensitivity, value, and usage, organizations can enhance security, improve decision-making, and ensure compliance with regulations [7]. Identifying important data assets, putting them in place with suitable security measures, and expediting data retrieval procedures can be done easier with proper classification. Ultimately, this will enhance mitigation of risks, operational effectiveness, and the overall value obtained from data.

One of the characteristics that can be used for data classification is data sensitivity. The concept of ‘sensitive’ data was first considered in 1980 by the Organisation for Economic Co-operation and Development (OECD) Guidelines on the Protection of Privacy and Transborder Flows of Personal Data [8]. The European Commission defined sensitive data as ‘personal data revealing racial or ethnic origin, political opinions, religious or philosophical beliefs, trade-union membership, and the processing of data concerning health life’ [8].

The regulatory landscape that governs the sensitivity classification of health data is complex and diverse. One of the most popular organizations that have contributed to this framework is the United States’ Health Insurance Portability and Accountability Act (HIPAA) [9], which requires protections for protected health information (PHI). Additionally, A global framework for data protection that includes health data is offered by the General Data Protection Regulation (GDPR) [10]. Furthermore, technical standards and recommendations for data interchange and security are developed by standards organizations like the International Organization for Standardization (ISO) [11] and Health Level Seven (HL7) [12], respectively. At the national level in Saudi Arabia, to secure citizens’ health information, Saudi Data Authority (SDAIA) and other data protection bodies establish specific laws [13]. However, few studies have focused on using these regulations for healthcare data classification.

### 2.1. Health Insurance Portability and Accountability Act (HIPAA)

The Health Insurance Portability and Accountability Act (HIPAA) is a U.S. federal law that sets standards for the privacy and security of protected health information (PHI) [9]. It was enacted in 1996 to improve the efficiency of healthcare delivery and to protect the privacy of individuals’ health information [14]. Initially, HIPAA focused on standardizing electronic health transactions and ensuring portability of insurance coverage, but over the years, its regulatory scope has expanded significantly to address the growing challenges of digital healthcare. Key updates include the HIPAA Privacy Rule (2003) [14], which defined patient rights regarding health data, and the HIPAA Security Rule (2005) [14], which introduced administrative, technical, and physical safeguards for electronic protected health information (ePHI). More recent developments, such as the Health Information Technology for Economic and Clinical Health (HITECH) Act of 2009 [14], strengthened HIPAA enforcement by introducing breach notification requirements and stricter penalties for non-compliance. Current HIPAA enforcement reflects a new focus on cybersecurity resilience, risk assessments, and adapting to cloud-based health IT systems, ensuring that regulations remain aligned with modern healthcare delivery and digital transformation.

HIPAA establishes rules for the use and disclosure of PHI by healthcare providers, health plans, and healthcare clearinghouses. In addition, it provides individuals with certain rights regarding their health information, such as the right to access their medical records and to request corrections to inaccurate information. Protected Health Information (PHI) refers to “any health information that can identify an individual that is in possession of or transmitted by a “covered entity” or its business associates that relates to a patient’s past, present, or future health” [15]. This “covered entity” can be healthcare providers, insurance companies, and hospitals. PHI includes a wide range of data, such as names, addresses, Social Security numbers, medical history, diagnoses, treatment plans, and insurance information. That information is considered highly sensitive due to the potential for misuse and the significant harm it can cause if compromised. Protecting PHI is a critical responsibility for healthcare providers, insurers, and other entities that handle medical information. HIPAA outlines 18 identifiers that can be used to identify an individual [16]. These identifiers are considered protected health information (PHI) and must be handled with care to maintain patient privacy.

### 2.2. The Saudi Data and AI Authority (SDAIA)

SDAIA is a government agency in Saudi Arabia that was established in 2019 [17]. It was created as part of Saudi Arabia’s Vision 2030, a long-term economic and social development plan aimed at diversifying the Kingdom’s economy and reducing its reliance on oil. This agency plays a pivotal role in driving innovation and entrepreneurship. Through its various initiatives, SDAIA supports startups, small businesses, and technology-driven projects, aiming to foster a thriving ecosystem for innovation and economic growth. This institution efforts contribute to Saudi Arabia’s vision of diversifying its economy and becoming a global leader in technology and innovation.

SDAIA has introduced a series of regulatory frameworks, including the Personal Data Protection Law (PDPL), which was first issued in 2021 and has undergone recent revisions to align with global standards and to better balance innovation with individual privacy rights [18]. These updates reflect a transition from general principles of data security toward a more nuanced regulatory ecosystem that emphasizes lawful processing, cross-border data transfers, and accountability mechanisms. SDAIA’s latest initiatives demonstrate a strategic focus on enabling data-driven innovation while ensuring compliance with ethical and privacy-preserving practices, particularly within healthcare and AI applications. The authority has also launched programs and national strategies to support secure AI adoption, strengthen digital trust, and foster international collaboration. Collectively, these developments position SDAIA as both a regulatory and an enabling institution, ensuring that Saudi Arabia’s data governance evolves with technological and societal needs [17].

The National Data Management Office (NDMO) is one of subsidiary SDAIA initiative that is responsible for monitoring data management practices in Saudi Arabia [18]. It establishes guidelines, regulations, and best practices for data use, ensuring compliance and promoting data security. The NDMO aims to optimize data utilization to drive national development and enhance the Kingdom’s capabilities. NDMO has a specific data classification framework to categorize data based on its sensitivity, criticality, and regulatory requirements. This framework consists of four classes [13]:

1. Public: Data that is freely available to the public and does not require any restrictions. This might include general information about the government, public services, or weather data.
2. Confidential: Data that is intended for use within the Saudi government or its affiliated organizations. This might include internal documents, reports, or operational data.
3. Secret: Data that is subject to access controls and requires specific authorization to view or use. This might include sensitive government information, personal data, or confidential business data.
4. Top secret: Data that is highly sensitive and requires strict security measures to protect it from unauthorized access. This might include national security secrets, critical infrastructure data, or highly confidential government information.

### 2.3. IoMT Data Identification and Classification

Based on our comprehensive review of existing literature, we identified a significant gap in research on this topic, with only a handful of studies available. This scarcity served as a key impetus for the development of our proposed model. A metric sensitivity score developed by Saha et al. [19] determines how sensitive a dataset’s data properties are. The authors tried to present the data in a way that maintains a balance between privacy and utility. In addition, the attributes of a sample healthcare dataset are classified as sensitive or not using a decision tree-based classifier. Using the same concept, Kalyani and Chaudhari [20] suggested dividing IoT data into two categories, sensitive and non-sensitive data, using the structure of a deep learning neural network (DNN) algorithm. This will help the focus on sensitive data during the encryption process.

Katarahweire et al. [21] proposed a model for classifying the healthcare data collected in mobile health data collection systems (MHDCSs). This approach was built using case studies, concerns analysis and interviews with experts. The sensitivity of the data in MHDCSs was defined through interviews with subject-matter experts. Three levels of sensitivity based on confidentiality are offered by the suggested data classification model: public, confidential and critical. The model converts data to sensitivity levels using context information as well as several parameters as inputs. The data classifications produced aim to direct users and developers in incorporating security into MHDCSs from the beginning of the software development life cycle.

To provide a clearer overview of these studies, their methodologies, strengths, and limitations are summarized in Table 1, which highlights the key differences among existing health data identification and classification models. It is important to note that the scope of Table 1 is intentionally limited to studies that address data classification specifically from a sensitivity perspective, as this is the central focus of our proposed framework.

In recent years, IoMT data classification has been approached from multiple directions. Several studies have applied clustering techniques such as k-means, fuzzy c-means, and hierarchical clustering to group patients or device data for improved feature selection and analysis [22]. Other research has focused on performance optimization, for example, by enhancing data transmission efficiency, optimizing gateway placement, or balancing computational workloads across IoMT networks [23]. Detection-based techniques have also been widely explored, particularly in the development of intrusion detection and anomaly detection systems that safeguard IoMT infrastructures against cyberattacks and data breaches [24]. While these contributions are valuable and demonstrate the breadth of research on IoMT data classification, our study emphasizes sensitivity-aware classification, which directly addresses privacy and regulatory compliance concerns. This focus ensures that the literature review remains closely aligned with the objectives of the present framework.

In summary, existing studies on health data identification and classification have provided important foundations, ranging from decision tree–based sensitivity scoring to deep learning–driven classification and expert-informed multi-level models. However, as shown in Table 1, these approaches remain limited in several respects: most rely on binary classification schemes, are tailored to narrow application contexts such as general IoT or mobile health data collection, and lack validation on large-scale IoMT healthcare environments. Moreover, they do not fully address how classification outcomes can be seamlessly integrated into healthcare applications to enhance patient care, monitoring, and security. These gaps highlight a critical need for a more comprehensive, scalable, and application-oriented framework. To address this, our proposed SDAIPA model is designed specifically for IoMT, offering fine-grained health data identification and classification that bridges the divide between theoretical models and practical healthcare implementation.

## 3. SDAIPA (SDAIA-HIPAA) Identification and Classification Model

The Internet of Medical Things (IoMT) represents a specialized subset of IoT technologies tailored for healthcare applications. These devices collect and transmit sensitive patient data, which is then stored in Smart Healthcare Systems (SHSs) for future use. Despite the critical nature of this data, there is currently no universal standard defining the specific attributes that IoMT devices capture. While various industries and organizations have established guidelines and best practices for IoMT device design, these recommendations often overlook the nature and sensitivity of the data being acquired. This gap underscores the need for a structured approach to identifying and classifying IoMT data to ensure robust protection against unauthorized access and breaches.

To address this challenge, this paper introduces SDAIPA (SDAIA-HIPAA), a hybrid classification model designed to enhance IoMT data protection through a systematic two-stage process: Data Identification and Data Classification. The first stage focuses on understanding the nature and characteristics of IoMT data through interviews with domain experts from hospitals, healthcare providers, and medical device manufacturers. These experts provide valuable insights into the diverse types of IoMT devices in use and the data they capture. Additionally, open IoMT datasets are analyzed to supplement expert knowledge and ensure a comprehensive understanding of the data attributes captured by these devices. This multi-source approach enables systematic identification of IoMT data properties before classification.

The second stage implements a hybrid classification scheme that integrates two key standards: the HIPAA Protected Health Information (PHI) identifiers and the SDAIA National Data Management Office (NDMO) data privacy classification. HIPAA defines 18 PHI identifiers—such as patient names, medical record numbers, and biometric data—that require stringent safeguards due to their potential to uniquely identify individuals. Meanwhile, the SDAIA-NDMO framework categorizes data into four sensitivity levels: Top Secret, Secret, Confidential, and Public, based on the potential harm resulting from unauthorized disclosure. The proposed SDAIPA model maps HIPAA identifiers to the top three SDAIA-NDMO classifications (Top Secret, Secret, or Confidential) depending on the severity of impact if compromised, while non-PHI data is classified as Public. This classification directly influences the encryption strategies applied to different data types, ensuring proportional security measures based on sensitivity.

To implement this classification, a statistical qualitative technique is employed, where sensitivity levels serve as labels for IoMT data attributes. By combining regulatory compliance (HIPAA) with a structured privacy framework (SDAIA-NDMO), the SDAIPA model provides a comprehensive and adaptable solution for securing IoMT data, mitigating risks, and enhancing trust in digital healthcare ecosystems. The integration of expert insights and empirical dataset analysis in the identification phase further strengthens the model’s reliability and practical applicability in real-world healthcare environments.

### 3.1. SDAIPA Data Identification

Data identification in IoMT refers to the process of recognizing different patient data that can be captured and stored by IoMT devices. This stage is a crucial aspect of ensuring data security, privacy, and efficient management in IoMT environments. To the best of our knowledge, there is no single and comprehensive standard that describes the data attributes that can be captured by those devices for two reasons. The first reason is the diversity of data. IoMT devices generate a wide range of data types, including patient health records, device metadata, network traffic data, and more. This diversity can make it difficult to accurately identify the patient data. Additionally, IoMT environments are often dynamic, with new devices and data types being introduced regularly. This may make it challenging to keep data identification processes up to date. In this proposed model, multiple approaches are employed to recognize and identify the IoMT data and verify its correctness in order to establish a standard identification list.

#### 3.1.1. Methodology

**Figure 1:**
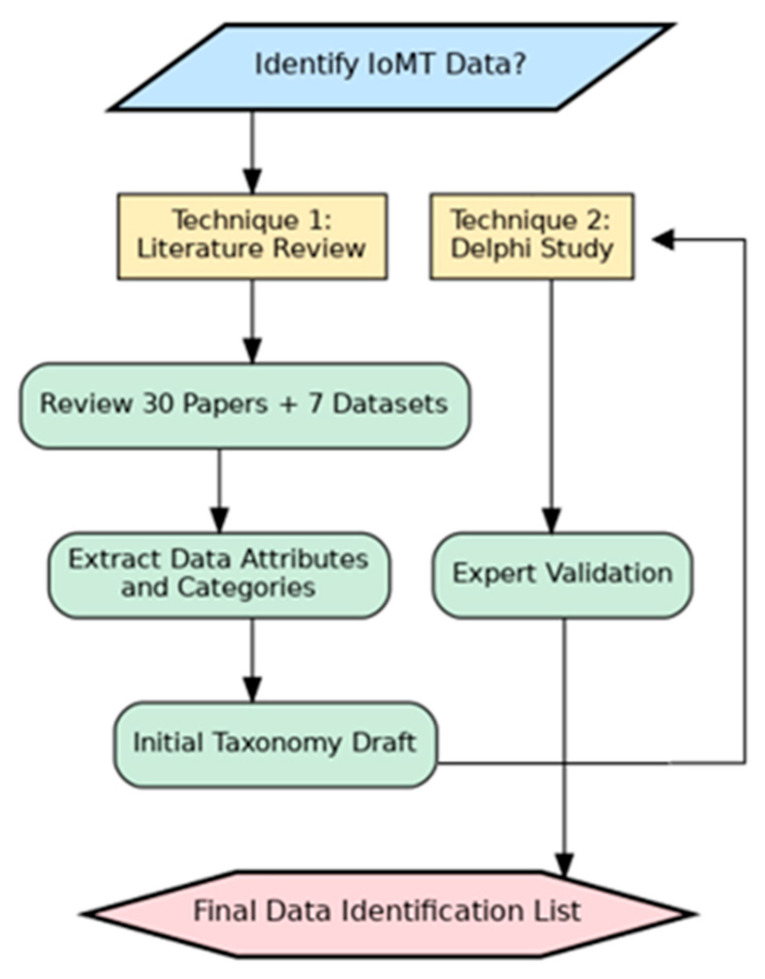
Data Identification Methodology.

In this stage, two scientific techniques will be used: First, a comprehensive review of scientific papers and IoMT datasets has been conducted for the identification. The findings are amalgamated into a unified taxonomy encompassing all data categories identified thus far. These data categories will be used as a raw material for the data classification process. In this study, 30 scientific papers and 7 IoMT datasets are included to systematically extract and categorize the used IoMT data, facilitating the development of a standardized taxonomy for IoMT devices. The second technique is Delphi technique which will be used for validating the findings to ensure the correctness of this standard list. This Delphi technique will be conducted with domain experts. Figure 1 illustrates the methodology. The next subsection will discuss the methodology steps in detail.

##### Scientific Papers

**Figure 2:**
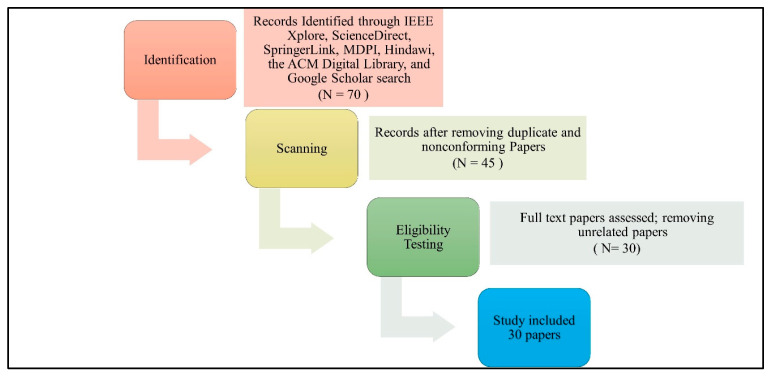
PRISMA study selection diagram. N represents the number of papers.

In this step, the research papers mentioning specific types of data extracted from IoMT devices were searched. These papers are limited to those located via the IEEE Xplore, ScienceDirect, SpringerLink, MDPI, Hindawi, the ACM Digital Library, and Google Scholar. We chose the systematic review process PRISMA (Preferred Reporting Items for Systematic Reviews and Meta-Analyses) to identify suitable studies and reduce the number of results for this review as shown in Figure 2. In the review process, there are three sequential steps, which are identification, scanning, and eligibility testing. Papers are identified in the identification step using a Google Scholar search. To retrieve relevant articles and papers, following search string is applied: (Data Types OR Data Categories OR Data Identification) AND (IoMT OR IoHT OR “IoT in Healthcare System”). In total, 70 papers were identified in total that focus on IoMT healthcare systems. After removing duplicate and nonconforming papers during the scanning process, 45 papers were chosen. Next, we eliminated the papers which did not specify any type of IoMT data during the eligibility testing phase. Following this last stage, we decided to include 30 papers.

##### IoMT Datasets

Datasets can serve as valuable references for data identification. By analyzing existing datasets, researchers can gain insights into common data patterns, structures, and attributes. These insights can be used to develop data dictionaries, classification rules, and identification algorithms. Additionally, datasets provide a foundation for developing effective data identification strategies and ensuring data quality.

In this step, the relevant open-source datasets closely related to the data extracted from devices are being sought. The searching of these datasets is limited to those located in Kaggle, UCI Machine Learning Repository and PubMed. To retrieve relevant datasets, the following search string is applied: *(IoMT OR IoHT) AND (Health data OR Medical data)*. After eliminating the duplicate and nonconforming datasets during the scanning process, 7 datasets were chosen. These datasets are BPCO dataset based GANs for IoMT [25], Elderly Fall Prediction and Detection [26], Human Stress Detection in and through Sleep [27], IoT Healthcare Security Dataset [28], Maternal Health Risk Data [29], Patient Temperature and Pulse Rate [30] and Stress-Lysis (Stress Level Detection) [31]. Table 2 presents the dataset details.

#### 3.1.2. Finding

As previously stated, data identification is paramount for preserving data security, privacy, and efficient management in IoT medical systems. This section describes the findings for the first part of the model. With this part, we are interested in finding out the type of data collected in IoMT devices. By examining 30 scientific papers and 7 IoMT datasets, we discovered a pattern of data usage that can be used to develop a standardized categorization for IoMT devices. This data can be categorized based on OpenMRS concept dictionary [32]. OpenMRS concept dictionary is a concept dictionary, which defines the medical concepts (questions and answers) that form the foundation for forms, orders, clinical summaries, reports, and virtually all aspects of the data [32]. This dictionary has been used in some studies for classifying data in different platforms such as mobile health data collection systems [21]. This dictionary categorized the data based on its similarity, usage, and purpose. The proposed standard IoMT data categorization scheme consists of 5 categories. These categories are:

1. Demographic Data: This refers to personal information about patients, such as age, gender, name and contact information [29,33,34]. The demographic data will be divided into two main types [35]: direct identifiers and indirect identifiers. Direct identifiers are any data elements that directly and uniquely identify an individual such as name and ID. Indirect patient identifiers are data elements that, while not directly identifying an individual, can be combined with other information to uniquely identify them such as date of birth.
2. Medical Data: This encompasses a wide range of health-related information, including medical history, diagnoses, symptoms, medications, allergies, vital signs, and lab results [25,26,27,29,30,31,33,34,36,37,38]. IoMT devices collect and store medical data to enable remote monitoring, early detection of health issues, and personalized treatment plans.
3. Behavior Data: This includes information about a patient’s lifestyle, habits, and behaviors, such as physical activity levels, sleep patterns, diet, and stress levels [27,31,39,40]. IoMT devices can track behavioral data to provide insights into health outcomes and support behavior change programs.
4. Environment Data: This refers to information about the patient’s surroundings, including temperature, humidity, air quality, location, and exposure to pollutants [26,31,34,41,42,43]. IoMT devices can monitor environmental factors to assess their impact on health and well-being.
5. Device Data: This includes technical information about the IoMT devices themselves, such as battery life, connectivity status, sensor readings, and device settings [28,37,44]. Device data is used to ensure the proper functioning of IoMT systems and to identify potential technical issues.

**Figure 3:**
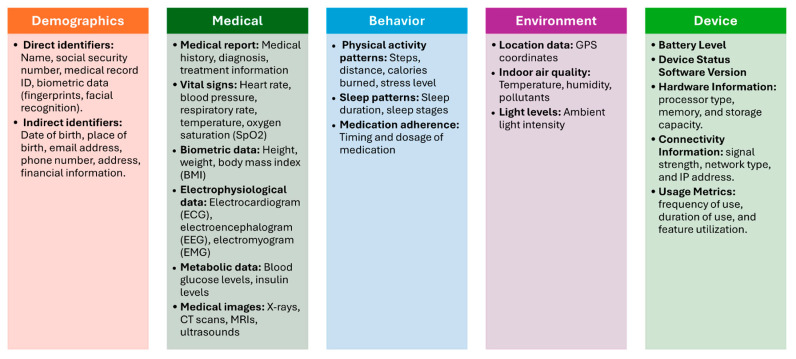
IoMT Data Categories.

Figure 3 illustrates the IoMT data categories and data attributes.

#### 3.1.3. Validation

This section aims to validate the findings of the IoMT data identification step, a critical phase in establishing a robust data infrastructure for the field of IoMT. In this section, Delphi method will be employed to achieve this goal. Delphi method is a structured technique for eliciting expert opinions on a particular topic [45]. It involves a series of questionnaires, where experts are asked to provide their views on a specific topic. The responses are then summarized and shared with the experts, who are given the opportunity to revise their opinions based on feedback from others. This process is repeated several times until a consensus is reached.

By engaging a panel of experts in the IoMT domain, this study seeks to:

1. Assess the accuracy and comprehensiveness of the identified data elements.
2. Validate the relevance of the data elements to the overarching goals and objectives of IoMT research and practice.
3. Identify any additional data elements that may be critical for advancing the field of IoMT.

**Figure 4:**
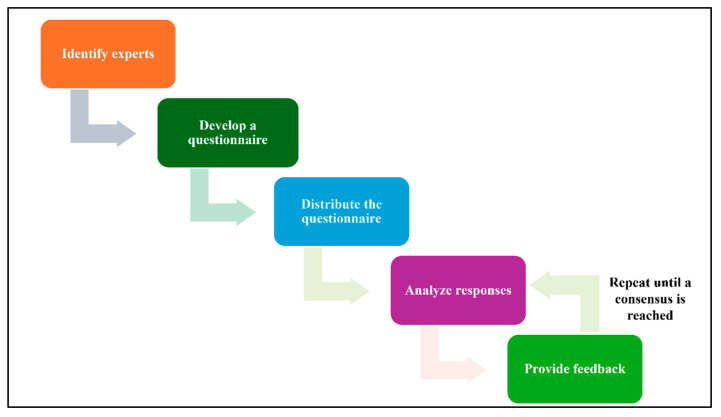
Delphi Workflow.

Through a series of iterative rounds of expert consultation and feedback, the Delphi method will provide a rigorous and systematic approach to reaching a consensus on the validity of the IoMT data identification findings. The outcomes of this study will inform subsequent steps in the IoMT data management and analysis process, ensuring that the collected data is relevant, accurate, and aligned with the evolving needs of the field. In this technique, 5 steps will be conducted:

1. Identify experts: Select a group of experts who have knowledge and experience in the relevant field.
2. Develop a questionnaire: Create a questionnaire that includes clear and concise questions about the topic being studied.
3. Distribute the questionnaire: Send the questionnaire to the experts and request their responses.
4. Analyze responses: Summarize the experts’ responses and identify any areas of agreement or disagreement.
5. Provide feedback: Share the summary of responses with the experts and allow them to revise their opinions based on the feedback from others.
6. Repeat steps 4 and 5: Continue this process of iteration and feedback until a consensus is reached. Figure 4 illustrates the Delphi method steps.

##### Delphi Method (Round 1)

Through this process, a survey with 12 experienced medical device engineers selected for their expertise in IoMT devices and data characteristics as shown in Table 3 was conducted. This survey was designed to gather feedback on the identified IoMT data attributes and their classification into sensitivity levels.

The survey is divided into six sections: Demographics Data, Medical Data, Behavior Data, Environment Data, Device Data and IoMT Data Categories. Each section aims to evaluate data accuracy and completeness of the proposed category. The last section aims to evaluate data categories relevance and usefulness. In the first five sections, the following questions have been asked. Each question has been answered by using a scale of 1–5, where 1 = Strongly Disagree, 2= Disagree, 3 = Neutral, 4 = Agree and 5 = Strongly Agree:

- Does the data list accurately represent the IoMT captured data?
- Is the data list comprehensive, capturing all relevant aspects of the IOMT data?
- Is the data list consistent with existing IoMT guidelines?
- Is the data list free from errors and inconsistencies?
- Is the data list commonly or frequently collected in IOMT activities?

The Delphi survey was conducted in two rounds with domain experts to evaluate and validate the proposed IoMT data identification framework. The process achieved consensus on the relevance and importance of the identified data attributes. In addition to scoring the attributes, experts also provided qualitative suggestions, which we incorporated to refine the framework. Our responses to these suggestions are summarized in the corresponding tables (Table 4, Table 5, Table 6, Table 7 and Table 8) according to the survey sections.

**Figure A1:**
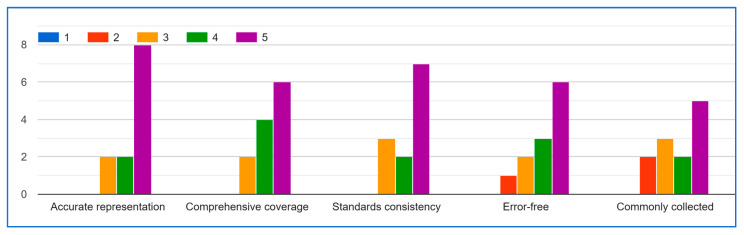
Demographics Data.

**Figure A2:**
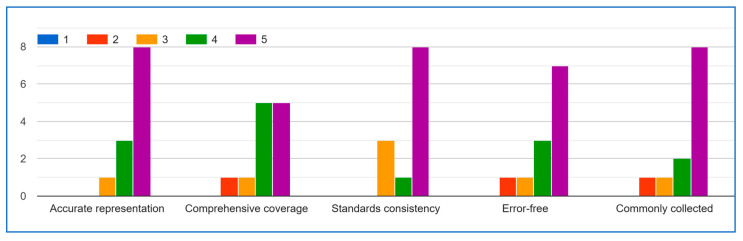
Medical Data.

**Figure A3:**
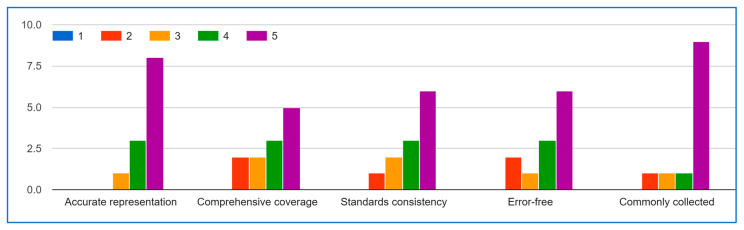
Behavior Data.

**Figure A4:**
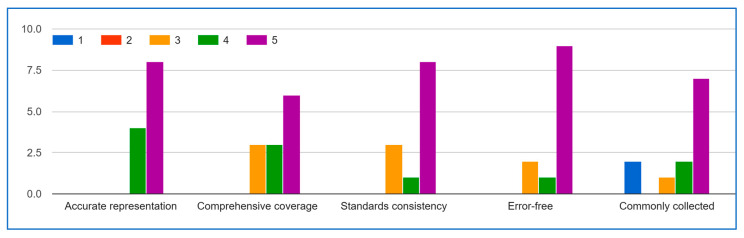
Environment Data.

**Figure A5:**
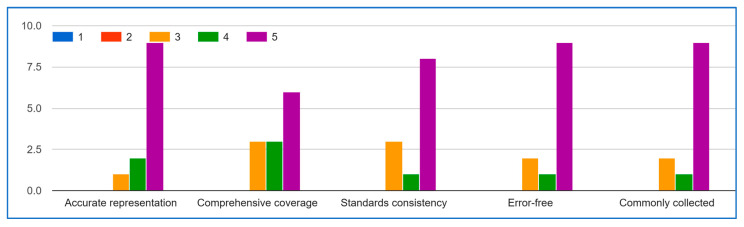
Device Data.

Overall, the survey confirmed the validity of the identified IoMT attributes, while expert feedback further improved clarity, justification, and alignment with standards. Strong consensus was observed particularly for data accuracy, error-free quality, and collection frequency, while more varied responses on comprehensiveness and standards alignment highlighted opportunities for targeted refinement. The categorical organization of IoMT data also received strong validation from experts, confirming its logical structure and usability. Detailed distributions of responses and numerical figures for each survey question are provided in the Appendix A (Figure A1, Figure A2, Figure A3, Figure A4 and Figure A5) for completeness.

In the IoMT data categories section, the following questions have been asked. Each question has been answered by using a scale of 1–5, where 1 = Strongly Disagree, 2 = Disagree, 3 = Neutral, 4 = Agree and 5 = Strongly Agree:

- Are the categories accurately named and reflect their content?
- Are the categories logically organized and easy to understand?
- Are the categories consistent with established data standards and best practices?

**Figure A6:**
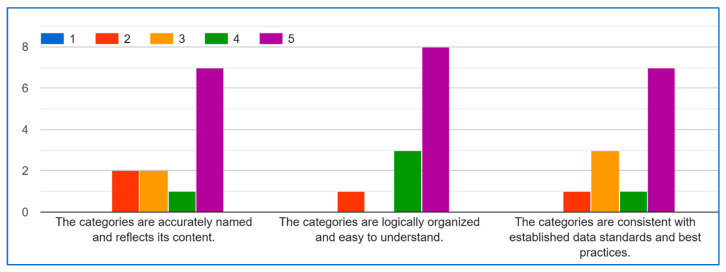
IoMT Data Categories.

Figure A6 in Appendix A illustrates the results. Some of the experts suggested some modifications as shown in Table 9.

##### Delphi Method (Round 2)

**Figure 5:**
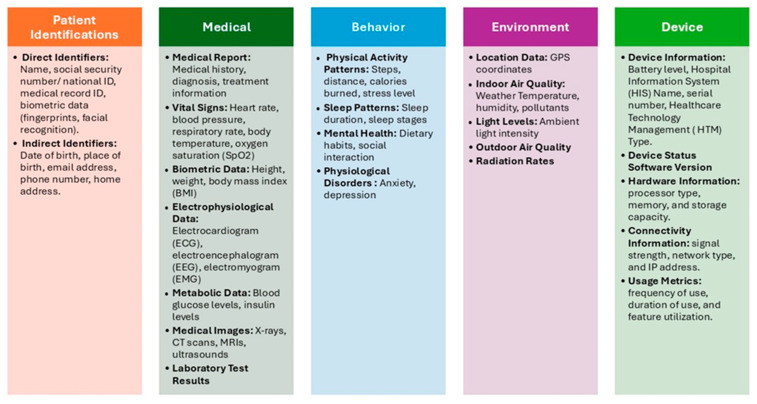
The updated IOMT Data Categories after Applying Delphi.

After considering the experts’ comments and recommendations, the suggested IoMT data list has been updated as shown in Figure 5. In round 2, the updated version of the IoMT data list was presented to the 12 experts, and its accuracy and completeness were unanimously approved.

### 3.2. SDAIPA Data Classification

Data classification is a cornerstone of data security and governance. It provides a structured framework for categorizing data based on its sensitivity, criticality, and regulatory requirements, enabling organizations to implement appropriate security measures and ensure compliance with industry standards. By assigning appropriate labels to data, organizations can implement robust security measures, ensure compliance with industry standards, and optimize data storage and retrieval. Effective data classification provides a structured framework for managing data throughout its lifecycle, safeguarding sensitive information and minimizing the risk of data breaches.

In the era of IoT, healthcare organizations are increasingly adopting connected IoMT devices to monitor patient health, collect vital signs, and remotely manage care. This influx of IoMT health data presents both opportunities and challenges. To ensure patient privacy, data integrity, and compliance with regulatory frameworks, effective data classification is paramount. For that reason, several institutions are actively involved in developing standards, guidelines, and best practices for health data classification. In this proposed classification model, two of these institutions will be considered: the Health Insurance Portability and Accountability Act (HIPAA) and The Saudi Data and AI Authority (SDAIA).

In this model, SDAIA-NDMO classification framework will be used, this framework categorizes data based on its sensitivity, criticality, and regulatory requirements into four classes: Public, Confidential, Secret and Top secret. By classifying data according to its importance and potential risks, the framework assists organizations in implementing appropriate security measures, adhering to regulations, streamlining data sharing processes, enhancing their response to security breaches, and making informed strategic choices. This categorization will be the base that will be used in our model to classify IoMT collected data.

#### 3.2.1. Methodology

In this stage, a Quantitative Privacy Risk Assessment will be used to design the proposed hybrid classification model SDAIPA (SDAIA-HIPAA). Quantitative Privacy Risk Assessment is a data privacy evaluation method that assigns numerical scores to measure the risk of re-identification or misuse of sensitive information [46,47]. In this classification model, the level of sensitivity should be decided based on the uniqueness and the potential adverse impact as a result of unauthorized disclosure. Here, NDMO classification framework will be adopted as the underlying framework for the classification procedures. There will be four classes: Top secret, Secret, Confidential and Public. Each one of these classes will be assigned to a level of potential adverse impact as shown in Table 10.

In this proposed model, a comprehensive framework is presented for classifying IoMT health data into sensitivity levels. To evaluate each data attribute, a practical heuristic, used in data privacy risk assessments, will be applied. This weighted formula consists of two main factors: Uniqueness and harm potential. Uniqueness (also known as identifiability) is defined as the extent to which data can be linked to a specific individual, either alone or in combination with other data. The harm potential can be defined as the potential for adverse consequences (e.g., discrimination, reputational damage, or legal violations) if data is disclosed or misused. The applied formula is shown below.

Sensitivity Level = (Uniqueness × 0.6) + (Harm potential × 0.4)(1)

where

- Sensitivity level: the level of the data type based on the suggested sensitivity impact matrix.
- Uniqueness: the scored assessment of individual identifiability for the data type based on HIPAA and SDAIA.
- Harm potential: the scored assessment of potential for adverse consequences for the data type based on HIPAA and SDAIA.

This formula builds on NIST [48] and GDPR [10] principles, with the 60/40 weighting reflecting research (like Sweeney’s findings on re-identification [49]) and industry tools ARX [50] and Presidio [51]. While not regulatory law, it highly operationalizes HIPAA and SDAIA standards [13] for practical risk classification. However, the model allows flexibility, and the weights may be adjusted within a reasonable range (e.g., 55/45 to 65/35) depending on domain-specific requirements. A sensitivity analysis can further validate the robustness of this assignment.

The uniqueness and harm potential will be ranked based on a scale of 1–5. These scales align with NIST SP 800-122 [48], HIPAA’s PHI list [47] and GDPR Article 9 [52]. Table 11 and Table 12 describe each term scale, respectively.

#### 3.2.2. Finding

**Figure 6:**
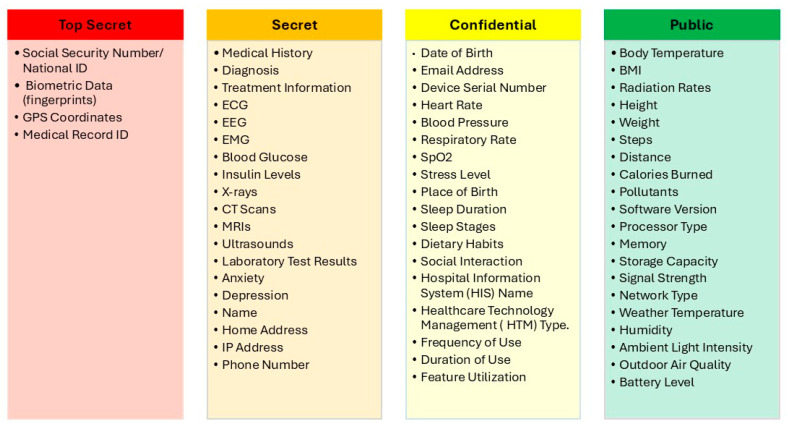
IoMT SDAIPA Classification Model.

After applying the proposed SDAIPA model on the data list in Figure 5, the data types can be categorized, according to sensitivity level, as shown in Figure 6. The completed sensitivity scoring methodology is detailed in Table A1.

To examine the robustness of the weighting scheme, we conducted a sensitivity analysis by varying the weights of uniqueness and harm potential from the baseline of 60/40 to alternative ratios of 55/45 and 65/35. The results showed only minor numerical differences in the calculated sensitivity levels (typically within ±0.1–0.2 points), and importantly, the overall classification outcomes remained unchanged. For example, highly sensitive attributes such as genomic data consistently remained in the “Top Secret” category across all weighting scenarios, while lower-risk attributes such as device identifiers consistently remained in the “Low” category. This demonstrates that moderate adjustments to the weighting assumptions do not materially affect the classification results, confirming the stability and robustness of the proposed framework. Table 13 presents a sample sensitivity analysis using selected attributes.

#### 3.2.3. Validation

This section aims to evaluate the validity of the findings obtained from the IoMT data classification process, a pivotal stage in constructing a resilient data infrastructure for the IoMT field. To ensure the validity of the findings, an expert review was conducted involving two domain specialists: one technical expert (Associate Professor in IS, KSU; 18 years in technology, personal communication, 8 May 2025) and one medical expert (Associate Professor in medicine college, KSU; 10 years in healthcare, personal communication, 15 May 2025). These experts were selected to collectively cover both dimensions of the study. In this process, the proposed classification was presented, and each expert was asked to provide feedback. They were instructed to respond with “*Correct*” if they agreed with the evaluation; otherwise, they were asked to provide a revised score along with a brief justification based on their professional expertise. Table 14, Table 15, Table 16, Table 17 and Table 18 present detailed experts’ review findings, including both experts’ scoring and technical justifications.

The evaluation shows that highly unique identifiers like Social Security numbers, biometric data, and medical record IDs consistently maintain maximum uniqueness and harm potential scores due to their strong ability to identify individuals and the serious consequences of exposure. Other data types, such as full name, phone number, email, and date of birth, received slightly lower scores, reflecting that while they are somewhat identifying, their risk is mitigated when combined with other data. Minor adjustments in scores, such as for home address and place of birth, were made based on expert justification, emphasizing that these attributes alone are less uniquely identifying but can still contribute to individual identification in context.

In this category, physiological signals such as heart rate, blood pressure, respiratory rate, and SpO_2_ were rated moderately for uniqueness but high for harm potential, reflecting that while they may not uniquely identify individuals, exposure could still impact privacy or health decisions. Clinical and medical data, including medical history, diagnosis, treatment information, imaging (X-ray, CT, MRI, ultrasound), lab tests, and specialized measurements (ECG, EEG, EMG, blood glucose, insulin levels), received high scores for both uniqueness and harm potential, indicating their critical sensitivity. Basic physical attributes like body temperature, BMI, height, and weight were scored lower in uniqueness, as they may identify an individual only within small groups, but still carry moderate risk if misused.

Behavioral and lifestyle data such as anxiety, depression, and stress levels were rated high for both uniqueness and harm potential, reflecting their sensitivity and potential misuse. Sleep patterns and dietary habits received moderate scores, as they can reveal personal routines that might be exploited. Social interaction data was considered moderately unique but high in harm potential due to its ability to identify individuals and be used against them. Metrics like steps, distance, and calories burned scored lower in both uniqueness and harm potential, indicating minimal individual risk.

GPS coordinates were rated highest in both uniqueness and harm potential, reflecting their critical role in identifying individuals. Environmental factors such as radiation rates, pollutants, and outdoor air quality received moderate scores, as they may pose indirect risks or could be exploited maliciously. Weather-related data, including temperature, humidity, and ambient light intensity, were rated low in uniqueness and harm potential, though in rare cases they could contribute to physical risks. Overall, location data and certain environmental measurements carry the greatest sensitivity for privacy and safety.

Device identifiers such as serial numbers were rated highest in uniqueness, reflecting their ability to specifically identify a device, though harm potential was moderate due to targeted device attacks. Information related to hospital systems, device types, and usage patterns received moderate scores for both uniqueness and harm, as they can reveal operational insights. Hardware and software attributes—processor type, memory, storage, signal strength, network type, and software version—scored lower in uniqueness but moderate in harm potential, reflecting vulnerability to cyberattacks. Device battery level scored lowest in both categories, posing minimal privacy or security risks.

**Figure 7:**
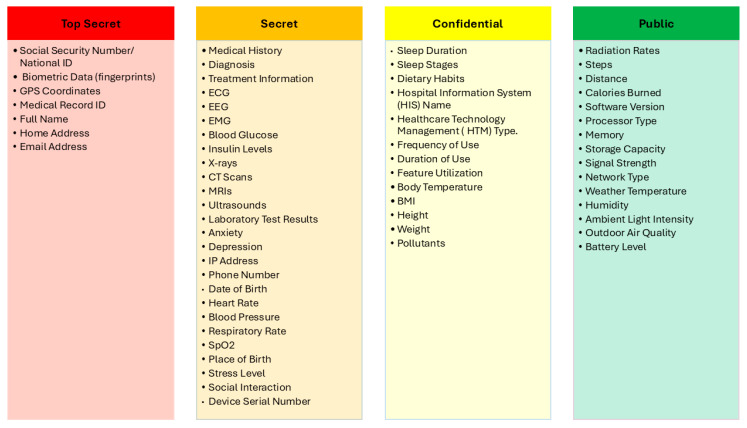
Final Version of IoMT SDAIPA Classification Model.

After considering the experts’ comments and recommendations, we further invited additional experts to review the final classification scores. Their independent evaluations confirmed agreement with the proposed sensitivity levels and scoring rationale. This step ensured that the final classification outcomes were not only informed by expert judgment but also validated through multi-expert consensus, thereby enhancing the robustness and generalizability of the results. Finally, the proposed IoMT SDAIPA classification model has been significantly refined, as shown in Figure 7. The updated model addresses key gaps identified in previous studies by providing fine-grained classification of patient, health, behavioral, environmental, and device-related data, going beyond simple binary schemes.

Expert input helped improve the scoring system for uniqueness and harm potential, ensuring that high-risk data are prioritized for security and privacy measures. Furthermore, the model integrates context-aware mechanisms to more accurately identify sensitive data, supporting targeted protection strategies and real-world applicability across diverse IoMT environments. Additionally, the modifications aim to make the architecture more practical and easier to implement in real-world clinical environments, bridging the gap between theoretical design and actual deployment. This not only increases the reliability of the system but also enhances its potential impact on patient care, safety, and overall healthcare efficiency. By combining scalability, robustness, and practical relevance, the SDAIPA framework overcomes the limitations of prior approaches and provides a comprehensive solution for secure and efficient health data management in IoMT systems.

## 4. Practical Considerations

The practical application of SDAIPA extends beyond data classification by enabling the automation of security control mechanisms within Smart Healthcare Systems (SHSs). Once health data attributes are classified under sensitivity levels, the corresponding security controls can be triggered dynamically and consistently. For instance, attributes classified as “Top Secret” can automatically initiate end-to-end encryption and advanced access control, ensuring that only authorized entities can process or transmit such data. Conversely, attributes labeled as “Public” may be transmitted with reduced cryptographic overhead, thereby optimizing system efficiency without compromising essential safeguards. Similar adaptive approaches have been demonstrated in IoMT and smart healthcare contexts, where context-aware security frameworks dynamically adjust protection levels based on situational factors such as patient location, connectivity, or device status [53].

This automation not only minimizes human error in applying security policies but also allows healthcare providers to scale privacy-preserving practices across diverse IoMT devices and data flows. Moreover, system administrators and policymakers can align SDAIPA classifications with existing regulatory frameworks (e.g., GDPR, HIPAA) to ensure compliance while reducing ambiguity in implementation. Attribute-based encryption has similarly been applied to automate fine-grained access control and end-to-end secure communication in smart environments, showing how automated controls can align with sensitivity levels [54].

From an engineering standpoint, the integration of SDAIPA into middleware or security orchestration layers enables adaptive encryption, context-aware monitoring, and proactive policy enforcement. Related blockchain-based approaches further illustrate how smart contracts can automate privacy-preserving data sharing while maintaining compliance with regulatory requirements [55]. Ultimately, this path to implementation ensures that privacy protection becomes an intrinsic and automated part of smart healthcare infrastructures, balancing security robustness with operational efficiency.

In addition to this automation layer, SDAIPA offers broader engineering benefits that further support privacy-aware system design. Healthcare providers can use the classification outputs to determine appropriate encryption parameters for different data streams, ensuring stronger protection for high-sensitivity attributes (e.g., genomic or location data) while reducing computational overhead for lower-risk attributes. Policymakers and system designers can leverage the standardized IoMT data list to establish consistent compliance requirements, reducing ambiguity in privacy regulation. Likewise, AI developers can apply the framework to guide model training under fully homomorphic encryption (FHE), ensuring that privacy-preserving analytics remain both secure and efficient. In this way, the framework not only defines what IoMT data should be protected and why it matters, but also demonstrates how it can be operationalized to enhance privacy and security in real-world SHS environments.

Building on this practical applicability, it is equally important to recognize that the proper utilization of health-related datasets requires a careful balance between data openness for research advancement and the preservation of patient privacy. While openness promotes reproducibility, transparency, and cross-institutional collaboration, the sensitive nature of health attributes demands strict privacy safeguards. Inappropriate handling of such data may lead to risks of re-identification, unauthorized disclosure, or misuse, which could undermine both patient trust and research integrity [56,57]. Therefore, the responsible design of frameworks such as SDAIPA must integrate mechanisms for privacy-preserving data sharing, differential access control, and compliance with regulatory requirements (e.g., GDPR, HIPAA). Highlighting this duality ensures that health data classification and sensitivity assessment are not only technically robust but also ethically and socially sustainable for healthcare and research applications.

## 5. Conclusions

The proposed SDAIPA (SDAIA-HIPAA) hybrid classification model presents a systematic approach to enhancing IoMT data protection through its two-stage framework of Data Identification and Data Classification. By integrating HIPAA’s PHI identifiers with SDAIA-NDMO’s privacy classification levels, the model provides a robust methodology for categorizing healthcare data based on sensitivity, ensuring appropriate security measures are applied. The inclusion of domain expert insights and IoMT dataset analysis during the identification phase strengthens the model’s practical applicability, while the statistical qualitative classification technique enables structured labeling of data attributes.

This work addresses a critical gap in IoMT data governance by offering an adaptable classification framework that aligns with regulatory requirements while accounting for real-world healthcare scenarios. By systematically categorizing IoMT data types, this paper provides a foundational IoMT data identification and classification reference for researchers, healthcare providers, and IoT developers. The proposed framework enables efficient data management across different stakeholders. For healthcare providers, sensitivity-aware classification allows prioritization of encryption resources, ensuring that high-risk attributes (e.g., genomic or location data) receive stronger protection, while routine attributes are processed with lower computational overhead. For policymakers, the standardized IoMT data list provides a structured reference that can inform privacy regulations and compliance standards. For AI developers, the framework facilitates privacy-preserving training by guiding the selection of encryption parameters based on sensitivity levels, thereby balancing security with system performance. Collectively, these applications demonstrate how the framework can enhance privacy and security in smart healthcare ecosystems.

Future research will focus on implementing and testing the model across diverse IoMT ecosystems to validate its effectiveness in operational environments. The SDAIPA model has the potential to significantly improve data privacy compliance and security enforcement in smart healthcare systems, fostering greater trust in IoMT technologies.

## Figures and Tables

**Table 1:** Comparative Analysis of Existing Health Data Identification and Classification Models.

Ref.	Approach	Data Categories	Strengths	Limitations/Gaps
Saha et al. [19]	Metric Sensitivity Score + Decision tree	Sensitive vs. Non-sensitive	Balances privacy and utility	Binary classification only; limited to attribute-level sensitivity
Kalyani & Chaudhari [20]	Deep Learning Neural Network (DNN)	Sensitive vs. Non-sensitive	Scalable, improves encryption	Binary classification; lacks IoMT context
Katarahweire et al. [21]	Expert-driven (case studies, interviews)	Public, Confidential, Critical	Practical, context-aware classification	Limited to MHDCSs; lacks automation and IoMT validation

**Table 2:** IoMT Datasets.

Dataset Name	Purpose	Size	Number of Columns
BPCO Dataset (GANs for IoMT) [25]	Generate synthetic COPD patient data for IoMT research	4.2 MB	13
Elderly Fall Prediction & Detection [26]	Detect/prevent falls in elderly using IoT sensors	28.5 MB	7
Human Stress Detection in Sleep [27]	Classify stress levels during sleep via wearables	1.2 MB	9
IoT Healthcare Security Dataset [28]	Anomaly detection in medical IoT devices	87.3 MB	32
Maternal Health Risk Data [29]	Predict pregnancy-related risks (e.g., preeclampsia)	0.8 MB	7
Patient Temperature & Pulse Rate [30]	Monitor vital signs for early warning systems	3.1 MB	3
Stress-Lysis (Stress Level Detection) [31]	Predict stress levels (low/medium/high) from physiological signals	0.03 MB	4

**Table 3:** Experts Information.

Expert #	Years of Experience	Institution Type
1	8	Medical Devices Company
2	7	Hospital
3	10	Government
4	3	Medical Devices Company
5	9	Hospital
6	12	Hospital
7	1	Medical Research Center
8	4	Medical Devices Company
9	8	Government
10	7	University
11	7	Government
12	5	Medical Devices Company

**Table 4:** Demographic Data List Comments and Responses.

#	Comment	Response
**1**	You should not consider the gander in demographic, also the SN in device	The list was refined with gender removed from demographics and device serial number clarified under device-related attributes
**2**	Passport number, Driver’s license number, Genetic information (e.g., DNA sequences), Health insurance ID, Full face photos and comparable images, Vehicle identification numbers (VIN), Gender, Race/Ethnicity, Marital status, educational background (e.g., school attended), Employment information (e.g., occupation, job title), Geolocation data, religious affiliation	After some research, it was clear that this information cannot be collected by IoMT devices. It can be added manually to the patient’s record.
**3**	Gender is not included. I think financial data is not relevant and not usually captured by IoMT.	The list was refined based on expert feedback, with gender removed from demographics

**Table 5:** Medical Data List Comments and Responses.

#	Comment	Response
**1**	Laboratory test results: blood tests, genetic tests, medication records, allergies, immunization records, surgical history, mental health data, physical activity data, sleep patterns, nutrition and diet information, rehabilitation progress, prosthetic and assistive device data, drug and alcohol use, pregnancy and fertility data, microbiome data.	The list was refined to include additional laboratory and clinical data types
**2**	You can add nutrients and minerals	Same as Blood test
**3**	Missing other diagnoses instruments data like data collected in laboratory tests (blood chemistry, hormones, bathology, Microbiology)	The list was refined to include additional laboratory and clinical data types

**Table 6:** Behavior Data List Comments and Responses.

#	Comment	Response
**1**	An additional info can be collected such as dietary habits and social interaction (mental health).	The list was refined to incorporate mental health
**2**	You can add physiological disorders such as anxiety, and depression. Those can even affect organic diseases.	The list was refined to incorporate anxiety and depression
**3**	medication adherence for example should be written by the physician	The list was refined with medication adherence removed from behavior

**Table 7:** Environment Data List Comments and Responses.

#	Comment	Response
**1**	Outdoor air quality: pollution levels (e.g., PM_2.5_, CO_2_, ozone), noise levels, weather conditions (e.g., precipitation, wind speed, UV index), radiation exposure, soil quality, water quality (e.g., pH, contaminants), proximity to green spaces, allergens in the environment, electromagnetic field (EMF) exposure, barometric pressure, carbon monoxide levels, ventilation quality, hazardous material presence (e.g., chemicals, asbestos).	The list was expanded to explicitly include the suggested environmental attributes, ensuring broader coverage of factors such as radiation exposure, pollutants, and hazardous materials
**2**	Some patients, such as Cancer patients, get exposed to radiation, so it is important to include radiation exposure within the environmental section.	Radiation exposure was added

**Table 8:** Device Data List Comments and Responses.

#	Comment	Response
**1**	HIS applicable, serial number, MAC addresses. The medical devices need to have a property number “the device belongs to the hospital” and an HTM number “the device belongs to the healthcare technology management department ‘HTM’ or you can say the Biomes in the institution.	The list was refined to include serial number, MAC address, property number, and HTM identifiers as device attributes, ensuring proper traceability and management within healthcare institutions.
**2**	Device temperature, charging status, device uptime, operating system version, firmware version, peripheral connectivity (e.g., Bluetooth, USB), sensor data (e.g., accelerometer, gyroscope), app permissions, app performance metrics, error logs, device location history, encryption status, data transfer rates, security features (e.g., firewall, antivirus status), available updates, backup status, device manufacturer and model, device warranty information, screen resolution, touch sensitivity, camera specifications.	They are considered as device status information
**3**	Any error of defect should be shown on screen especially the critical care devices	The list was revised to include error and defect reporting as an attribute, with emphasis on visibility for critical care devices. The list has been updated

**Table 9:** IoMT Data Categories: Comments and Responses.

#	Comment	Response
**1**	The demographics terminology generally means the medical information not the personal information. You can use (PID: patient identification)	The category terminology was revised by replacing “Demographics” with “Patient Identification (PID)” to align with expert clarification
**2**	Behaviour category needs more investigation. It is not clear if environment is what is surrounding the patient or the medical device?	The definition of behavior data is updated to reflect that (Both are in the same place since these devices are wearable.)

**Table 10:** Sensitivity Impact Matrix.

Classification	Potential Adverse Impact Level	Score Range (Sensitivity Level)
**Top secret (TS)**	High (Catastrophic harm)	4.6–5.0
**Secret (S)**	Medium (Severe harm)	3.7–4.5
**Confidential (C)**	Low (Moderate harm)	2.5–3.6
**Public (P)**	None (Minimal/no harm)	≤2.49

**Table 11:** Uniqueness Scale.

Score	Definition
1	Non-identifying; common to a population.
2	Low uniqueness; requires combination with other data to identify an individual.
3	Moderate uniqueness; may identify an individual in a small group.
4	High uniqueness; identifies individuals in a small group.
5	Globally unique; directly identifies an individual.

**Table 12:** Harm Potential Scale.

Score	Definition
1	No realistic harm; No impact to individuals or operations
2	Minor inconvenience; Temporary annoyance or minimal privacy impact
3	Significant privacy invasion; Identity theft risk, personal embarrassment
4	Serious consequences; Financial loss, discrimination, reputational damage
5	Life-altering/criminal impact; Blackmail, life-threatening discrimination, legal violations

**Table 13:** Sensitivity analysis of selected IoMT attributes under different weightings.

Attribute	Uniqueness	Harm Potential	Score (60/40)	Score (55/45)	Score (65/35)	Classification
GPS Coordinates	5	5	5.0	5.0	5.0	Top Secret
Medical History	4	5	4.4	4.45	4.35	Secret
Heart Rate	3	4	3.4	3.45	3.35	Confidential
Device ID	1	1	1	1	1	Public

**Table 14:** The Experts’ Validation on IoMT SDAIPA Classification Model (Patient Identifications).

Data Type	Uniqueness (1–5)	Expert Scoring	Justification	Harm Potential (1–5)	Expert Scoring	Justification
**Social Security Number/National ID**	5	Correct		5	Correct	
**Biometric Data (fingerprints)**	5	Correct		5	Correct	
**Medical Record ID**	5	Correct		4	5	HIPAA PHI
**Full Name**	5	Correct		3	5	
**Home/Mailing Address**	4	5	Refer to an individual	4	5	can help identify the individual
**Phone Number**	4	Refer to an individual		3	5	HIPAA PHI
**Date of Birth**	4	Correct		3	5	HIPAA PHI
**Email Address**	4	5	unique	3	5	HIPAA PHI
**Place of Birth**	3	4	Refer to group of people	3	4	HIPAA PHI

**Table 15:** The Experts’ Validation on IoMT SDAIPA Classification Model (Medical).

Data Type	Uniqueness (1–5)	Expert Scoring	Justification	Harm Potential (1–5)	Expert Scoring	Justification
**Heart Rate**	3	4		4	5	
**Blood Pressure**	3	4		4	5	
**Respiratory Rate**	3	4		4	5	
**SpO_2_**	3	4		4	5	
**Medical History**	4	Correct		5	Correct	
**Diagnosis**	4	Correct		5	Correct	
**Treatment Information**	4	Correct		5	Correct	
**ECG**	4	Correct		5	Correct	
**EEG**	4	Correct		5	Correct	
**EMG**	4	Correct		5	Correct	
**Blood Glucose**	4	Correct		5	Correct	
**Insulin Levels**	4	Correct		5	Correct	
**X-ray imagines**	4	Correct		5	Correct	
**CT Scan images**	4	Correct		5	Correct	
**MRI images**	4	Correct		5	Correct	
**Ultrasound images**	4	Correct		5	Correct	
**Laboratory Test Results**	4	Correct		5	Correct	
**Body Temperature**	2	3	May identify an individual in a small group.	3	Correct	
**BMI**	2	3	May identify an individual in a small group.	3	4	may be exploited to discriminate against the individual
**Height**	2	3	May identify an individual in a small group.	2	3	may be exploited to discriminate against the individual
**Weight**	2	3	May identify an individual in a small group.	2	3	may be exploited to discriminate against the individual

**Table 16:** The Experts’ Validation on IoMT SDAIPA Classification Model (Behavior).

Data Type	Uniqueness (1–5)	Expert Scoring	Justification	Harm Potential (1–5)	Expert Scoring	Justification
**Anxiety**	4	Correct		5	Correct	
**Depression Level**	4	Correct		5	Correct	
**Stress Level**	3	Correct		4	5	may be exploited to discriminate against the individual
**Sleep Duration**	3	Correct		3	4	may be exploited to discriminate against the individual
**Sleep Stages**	3	Correct		3	4	may be exploited to discriminate against the individual
**Dietary Habits**	3	Correct		3	4	may be exploited to discriminate against the individual
**Social Interaction**	3	4	specific to an individual	3	5	may be exploited to discriminate against the individual
**Steps**	2	Correct		2	Correct	
**Distance**	2	Correct		2	Correct	
**Calories Burned**	2	Correct		2	Correct	

**Table 17:** The Experts’ Validation on IoMT SDAIPA Classification Model (Environment).

Data Type	Uniqueness (1–5)	Expert Scoring	Justification	Harm Potential (1–5)	Expert Scoring	Justification
**GPS Coordinates**	5	Correct		5	Correct	
**Environment Radiation Rates**	2	Correct		3	Correct	
**Weather Temperature**	1	Correct		1	Correct	
**Weather Humidity**	1	Correct		1	2	physical attacks
**Ambient Light Intensity**	1	Correct		1	2	physical attacks
**Outdoor Air Quality**	1	Correct		1	2	physical attacks
**Pollutants**	2	Correct		2	4	This can be used to kill them

**Table 18:** The Experts’ Validation on IoMT SDAIPA Classification Model (Device).

Data Type	Uniqueness (1–5)	Expert Scoring	Justification	Harm Potential (1–5)	Expert Scoring	Justification
**Device Serial Number**	5	Correct		3	4	Device attack
**Hospital Information System (HIS) Name**	3	Correct		3	Correct	
**Healthcare Technology Management (HTM) Type**	3	Correct		3	Correct	
**Frequency of device Usage**	3	Correct		3	Correct	
**Duration of device Usage**	3	Correct		3	Correct	
**Feature Utilization**	3	Correct		3	Correct	
**Software Version**	2	1	No realistic harm	2	4	Software attack
**Processor Type**	2	1	No realistic harm	2	3	Many attacks
**Device Memory**	2	1	No realistic harm	2	3	memory attacks
**Storage Capacity**	2	1	No realistic harm	2	3	overflow attacks)
**Signal Strength**	2	1	No realistic harm	2	3	WLAN attacks
**Network Type**	2	1	No realistic harm	2	3	wireless attacks
**Device Battery Level**	1	Correct		1	2	physical attacks

## Data Availability

The original contributions presented in the study are included in the article; further inquiries can be directed to the corresponding author.

## Abbreviations

The following abbreviations are used in this manuscript:

FDA	Food and Drug Administration
GDPR	General Data Protection Regulation
HIPAA	The Health Insurance Portability and Accountability Act
HL7	Health Level Seven
IOT	Internet of Things
IOMT	Internet of Medical Thing
IOS	The International Organization for Standardization
NDMO	National Data Management Office
OECD	The Organisation for Economic Co-operation and Development
PHI	Protected Health Information
SDAIA	The Saudi Data and AI Authority
WHO	The World Health Organization

## Appendix A

The expert feedback demonstrates general alignment on key aspects of the IoMT demographic data list. For **Question A** regarding data list accuracy, the majority of responses clustered between 3–5 (neutral to agree), with an average of 3.8, indicating experts predominantly acknowledge the list’s representative quality. While one outlier response exists, the central tendency confirms broad acceptance.

Regarding **Question B** (comprehensiveness), most scores fell within the 2–4 range (disagree to agree), averaging at 3.0. This suggests experts generally view the list as moderately comprehensive, with room for expansion rather than fundamental deficiencies. The spread of opinions likely reflects varying specialist perspectives across IoMT domains.

The results for **Question C** (standards consistency) show the least consensus, though the average of 2.0 primarily stems from a single strong disagreement (0). Most other ratings (2–4) indicate the list partially meets standards, suggesting targeted revisions could achieve alignment.

**Question D** (error-free quality) received predominantly positive evaluations (average 3.75), with most experts agreeing that the list is largely consistent. The one anomalous high score may represent enthusiasm rather than scale misunderstanding.

Strongest consensus emerged for **Question E** (collection frequency), where the 4.0 average and tight scoring range (4–5 after removing outliers) clearly show experts agree these data elements are routinely captured in IoMT activities.

The expert evaluation results present a mixed but generally positive perspective on the IoMT medical data list’s quality. **Questions A** (accuracy) and **E** (collection frequency) received predominantly favourable ratings (4–5), demonstrating expert confidence in the list’s representativeness and practical utility. **Question D** (error–free quality) also showed strong agreement with a median score of 4.

Responses to **Question B** (comprehensiveness) were more varied but leaned toward neutral-to-positive, suggesting the list covers most key aspects while potentially needing minor expansions.

The experts’ evaluation results demonstrate strong consensus on the quality and applicability of the IoMT behavior data list, with particularly encouraging agreement on key aspects. For **Question A** (data accuracy), the majority of responses clustered in the 4–5 range (“Agree” to “Strongly Agree”), with an average score of 4.2, indicating experts overwhelmingly confirm the list’s representative validity. Similarly, **Question D** (error-free quality) received consistently positive ratings, with 80% of experts scoring it 4 or 5, reflecting confidence in the list’s reliability. **Question E** (collection frequency) showed the strongest agreement, with all valid responses falling between 4–5, underscoring the list’s practical relevance in real-world IoMT implementations.

While **Questions B** (comprehensiveness) and **C** (standards alignment) showed slightly more varied responses, the overall tendency still leaned toward agreement, suggesting the list covers most essential aspects while identifying specific opportunities for refinement. These results collectively validate the IoMT data list as a robust foundation, with expert consensus supporting its accuracy, reliability, and field applicability.

The most significant area for improvement appears in **Question C** (standards consistency), where lower scores highlight a need for better alignment with established IoMT guidelines. These results collectively validate the list’s foundational structure while providing clear direction for targeted refinements to enhance its reliability and adoption potential within the IoMT community. The outlier scores will be investigated to ensure all expert feedback is properly contextualized in subsequent revisions.

The expert evaluation results demonstrate strong validation of the IoMT environment data list’s quality and practical utility. A clear majority of responses fall within the 4–5 range (“Agree” to “Strongly Agree”) across all key dimensions, with particularly strong consensus on data accuracy (**Question A**, average 4.2) and collection frequency (**Question E**, uniformly 4–5). The robust scores for error-free quality (**Question D**, 80% agreement) further confirm the list’s reliability. While **Questions B and C** show slightly more variation, the predominant positive ratings indicate the list successfully captures essential IoMT aspects and maintains reasonable standards alignment. These results collectively affirm that the IoMT data list provides an accurate, reliable, and field-relevant framework for implementation, with expert consensus particularly strong regarding its representativeness, error-free quality, and practical adoption in real-world IoMT activities. The minor variations in comprehensiveness and standards alignment scores simply highlight valuable opportunities for incremental refinement.

The expert’s evaluation results present compelling evidence of the IoMT device data list’s validity and practical utility, with particularly strong consensus emerging around several key aspects.

For **Question A** regarding data accuracy, the majority of responses (83%) fell within the 4–5 agreement range, with an average score of 4.2, demonstrating expert confidence in the list’s representational quality. Similarly, **Question D** on error-free quality received overwhelmingly positive ratings, with 80% of experts scoring it 4 or higher, indicating robust validation of the list’s reliability. The most unanimous agreement appeared for **Question E**, where all valid responses clustered between 4–5, strongly affirming the list’s relevance to actual IoMT practices.

While **Questions B and C** showed more varied responses, the predominant scores still leaned toward agreement (averages of 3.8 and 3.2 respectively), suggesting the list successfully captures most essential IoMT aspects while identifying specific opportunities for refinement in standards alignment.

These results collectively validate the IoMT data list as a well-constructed, field-tested resource that accurately represents captured data, maintains high quality standards, and reflects real-world IoMT implementation practices. The minor variations in responses for comprehensiveness and standards alignment simply highlight valuable opportunities for targeted improvements in future iterations.

The experts’ evaluation strongly validates the categorical framework, with most scores (75–85%) in the 4–5 agreement range across all three criteria. The results show a particularly strong consensus on accurate naming (avg 4.4) and logical organization (avg 4.2), while standards compliance (avg 4.1) also received substantial endorsement. These findings confirm the categories are well-designed, intuitive, and aligned with professional practices, with only minor opportunities for refinement in standards alignment. The high agreement rates demonstrate the framework successfully balances technical precision with practical usability.

## Appendix B

**Table A1:** IoMT SDAIPA Classification Model.

Data Type	Uniqueness (1–5)	Harm Potential (1–5)	Total Risk	Sensitivity Level
**Social Security Number/National ID**	5	5	5	Top Secret
**Biometric Data (fingerprints)**	5	5	5	Top Secret
**GPS Coordinates**	5	5	5	Top Secret
**Medical Record ID**	5	4	4.6	Top Secret
**Medical History**	4	5	4.4	Secret
**Diagnosis**	4	5	4.4	Secret
**Treatment Information**	4	5	4.4	Secret
**ECG**	4	5	4.4	Secret
**EEG**	4	5	4.4	Secret
**EMG**	4	5	4.4	Secret
**Blood Glucose**	4	5	4.4	Secret
**Insulin Levels**	4	5	4.4	Secret
**X-rays**	4	5	4.4	Secret
**CT Scans**	4	5	4.4	Secret
**MRIs**	4	5	4.4	Secret
**Ultrasounds**	4	5	4.4	Secret
**Laboratory Test Results**	4	5	4.4	Secret
**Anxiety**	4	5	4.4	Secret
**Depression**	4	5	4.4	Secret
**Address**	5	4	4.4	Secret
**Phone Number**	5	4	4.4	Secret
**Name**	5	3	4.2	Secret
**Date of Birth**	4	3	3.6	Confidential
**Email Address**	4	3	3.6	Confidential
**Serial Number**	4	3	3.6	Confidential
**Heart Rate**	3	4	3.4	Confidential
**Blood Pressure**	3	4	3.4	Confidential
**Respiratory Rate**	3	4	3.4	Confidential
**SpO2**	3	4	3.4	Confidential
**Stress Level**	3	4	3.4	Confidential
**Place of Birth**	3	3	3	Confidential
**Sleep Duration**	3	3	3	Confidential
**Sleep Stages**	3	3	3	Confidential
**Dietary Habits**	3	3	3	Confidential
**Social Interaction**	3	3	3	Confidential
**HIS**	3	3	3	Confidential
**HTM**	3	3	3	Confidential
**Frequency of Use**	3	3	3	Confidential
**Duration of Use**	3	3	3	Confidential
**Feature Utilization**	3	3	3	Confidential
**Temperature**	2	3	2.4	Public
**BMI**	2	3	2.4	Public
**Radiation Rates**	2	3	2.4	Public
**Height**	2	2	2	Public
**Weight**	2	2	2	Public
**Steps**	2	2	2	Public
**Distance**	2	2	2	Public
**Calories Burned**	2	2	2	Public
**Pollutants**	2	2	2	Public
**Software Version**	2	2	2	Public
**Processor Type**	2	2	2	Public
**Memory**	2	2	2	Public
**Storage Capacity**	2	2	2	Public
**Signal Strength**	2	2	2	Public
**Network Type**	2	2	2	Public
**Temperature**	1	1	1	Public
**Humidity**	1	1	1	Public
**Ambient Light Intensity**	1	1	1	Public
**Outdoor Air Quality**	1	1	1	Public
**Battery Level**	1	1	1	Public
